# Hyper Diversity, Species Richness, and Community Structure in ESS and Non-ESS Communities

**DOI:** 10.1007/s13235-025-00646-2

**Published:** 2025-05-30

**Authors:** Kailas Shankar Honasoge, Tania L. S. Vincent, Gordon G. McNickle, Roel Dobbe, Kateřina Staňková, Joel S. Brown, Joseph Apaloo

**Affiliations:** 1 Systems Decision Methods, Faculty of Technology, Policy, and Management, Delft University of Technology, 2628BX Delft, The Netherlands; 2 Anchorage, Alaska 99518 USA; 3 Department of Biological Sciences, University of Illinois at Chicago, 845 W. Taylor Street, Chicago, IL 60607 USA; 4 Engineering Systems and Services, Faculty of Technology, Policy, and Management, Delft University of Technology, 2628BX Delft, the Netherlands; 5 Department of Integrated Mathematical Biology and Cancer Biology and Evolution Group, Moffitt Cancer Center, Tampa, FL 33612 USA; 6 Department of Mathematics and Statistics, St. Francis Xavier University, Antigonish, NS B2G 2W5 Canada

**Keywords:** Hypersaturated communities, Non-ESS communities, Mutual invasibility, Niche coevolution, Darwinian Dynamics, Evolutionary game theory

## Abstract

In mathematical models of eco-evolutionary dynamics with a quantitative trait, two species with different strategies can coexist only if they are separated by a valley or peak of the adaptive landscape. A community is ecologically and evolutionarily stable if each species’ trait sits on global, equal fitness peaks, forming a *saturated* ESS community. However, the adaptive landscape may allow communities with fewer (*undersaturated*) or more (*hypersaturated*) species than the ESS. Non-ESS communities at ecological equilibrium exhibit *invasion windows* of strategies that can successfully invade. *Hypersaturated* communities can arise through *mutual invasibility* where each non-ESS species’ strategy lies in another’s invasion window. Hypersaturation in ESS communities with more than 1 species remains poorly understood. We use the *G*-function approach to model niche coevolution and Darwinian dynamics in a Lotka–Volterra competition model. We confirm that up to 2 species can coexist in a hypersaturated community with a single-species ESS if the strategy is scalar-valued, or 3 species if the strategy is bivariate. We conjecture that at most $n \cdot \left( {s + 1} \right)$ species can form a hypersaturated community, where $n$ is the number of ESS species at the strategy’s dimension $s$. For a scalar-valued 2-species ESS, 4 species coexist by “straddling” the would-be ESS traits. When our model has a 5-species ESS, we can get 7 or 8, but not 9 or 10, species coexisting in the hypersaturated community. In a bivariate model with a single-species ESS, an infinite number of 3-species hypersaturated communities can exist. We offer conjectures and discuss their relevance to ecosystems that may be non-ESS due to invasive species, climate change, and human-altered landscapes.

**Supplementary Information:**

The online version contains supplementary material available at 10.1007/s13235-025-00646-2.

## Introduction

Closely related, coexisting species generally differ in just a few ecologically relevant traits. For instance, eastern grey squirrels (*Sciurus carolinensis*) and eastern fox squirrels (*Sciurus niger*) of the Midwestern United States differ in body size (the former smaller than the latter species), coat color (the former with a variable grey coat and the latter with a much less variable orange coat), and temperament, with grey squirrels being more social and adept at interspecific competition and fox squirrels seemingly more adept at managing predation risks [30, 41]. For Darwin’s finches, beak length, beak depth and overall body size are the salient traits that allow coexisting species to partition niche axes of seed size and seed hardness [23, 40]. These examples illustrate the interplay between suites of continuous traits possessed by coexisting species competing for resources along one or several niche axes.

Evolutionary game theory can be used to model these systems. The traits of the related species can vary along continuous trait axes. These differences permit or preclude coexistence along the corresponding niche axes. Via ecological and evolutionary dynamics, these games of community organization model species diversity, coevolution and speciation [24, 29, 33]. Such games are usually built upon models from population ecology (e.g. Lotka–Volterra competition equations [9, 28, 43]), where some of the model parameters are modified as functions of the strategies of the individuals in a population. Here, we consider game theoretic strategies to be heritable phenotypes. In game theoretic models, a species can be defined as a group of individuals possessing the same strategy, different species are groups with different strategies. An evolutionary equilibrium of such models may contain a single evolutionarily stable strategy (ESS) or multiple ESS strategies forming stable communities of one or more species. How do the different species come to be in such models?

One approach is to let the community achieve evolutionary equilibrium by the combined effects of strategy and population dynamics, sometimes termed Darwinian dynamics [42] or adaptive dynamics [21, 31, 32]. Faunal buildup can occur as strategies evolve to a convergent stable minima of the adaptive landscape [8]. Such points have been termed evolutionarily stable minima [3] or branching points [21]. One can imagine adaptive speciation resulting from the disruptive selection that acts on these points [11, 16, 17, 39]. Eventually, the diversity of species and their strategy values may converge on one or multiple ESSs. Or, if not, then additional species can be added until the ecological and evolutionary dynamics acting on each species and its strategy converge on an ESS community. At this point, all species’ strategies reside on peaks of the adaptive landscape and no rare species with an alternative strategy can invade. The ESS community is thus diverse enough to resist invasion, but not so diverse to be unstable.

An alternative approach to adding diversity to a game of community structure is to introduce invading species with distinct yet evolutionarily fixed strategies. Such additions can grow, shrink or maintain the community’s diversity based on whether successful invasions result in the loss of none, one or more current species [12]. In the absence of evolutionary dynamics acting on the species’ strategies, species cannot evolve to adaptive peaks. However, they may still coexist or go extinct depending on competitive dynamics. The resulting communities will be non-ESS, in that species may reside near peaks of the adaptive landscape, but only coincidentally on a peak. In this way, a kind of co-evolutionary dynamics emerges either through (1) the success of an invader species with a novel strategy resulting in either the loss or addition to the existing species, or (2) existing species produce occasional, rare mutant species with strategies that may be close to or far from the progenitor species [13, 14]. If mutations around extant species are small and relatively frequent, an eco-evolutionary dynamic can emerge in which strategies climb the slopes of the adaptive landscape [19].

Thus, in game theoretic models of ecological communities, coexisting groups of species may possess the strategies of the ESS (ESS communities) or strategies that are close or far from the actual ESS (non-ESS communities). Real communities may exhibit the same. Perhaps some communities have had sufficient time, strategy diversification, and coevolution to achieve their ESS. Or, because of continual disturbances, invasions or extinctions, the diversity of species and their strategies are non-ESS. In fact, with the scale of human disturbance to communities around the world, few communities may be at their ESS. But, ecologically, do ESS and non-ESS communities really differ in ways that would be important or conspicuous to us?

Our goal is to explore this question using an evolutionary game based on *G*-functions formulated as Lotka–Volterra competition. We shall explore the community properties of this model when the number of species at the ESS varies from few to many, and when the trait space is one or two dimensional. We compare ESS communities to non-ESS communities. Of particular interest is an important result by Durinx et al. [18] and further explored by Rubin et al. [37], showing that more species can coexist within a non-ESS than an ESS community. We explore Durinx et al.’s Proposition 5 in more detail with an emphasis on its ecological implications for natural communities and invasive species ecology. We can use the Lotka–Volterra evolutionary game to make predictions for how ESS and non-ESS communities differ and how they might be identified in nature. This becomes important as we may expect non-ESS communities to exhibit rapid evolution towards fewer species, or quite different patterns of community structure.

## Background

In the *G*-function approach, the fitness (or per capita growth rate) of a focal individual depends on its own strategy $v$, as well as the strategies of other individuals in the population ${\varvec{u}}=({u}_{1},{u}_{2},\dots ,{u}_{n})$. It is also influenced by the population sizes corresponding to these strategies ${\varvec{x}}=({x}_{1},{x}_{2},\dots ,{x}_{n})$ where ${x}_{i}$ represents the population density of individuals with strategy ${u}_{i}$ and $n$ is the number of distinct strategies in the community. This relationship is captured by $G\left(v, {\varvec{u}}, {\varvec{x}}\right)$. For our purposes, we see distinct ${u}_{i}$’s as different species and thus $n$ represents current species richness. This function is analogous to the invader fitness function of adaptive dynamics [1, 2, 25].

Using Darwinian dynamics, the changes in the population size of species *i* can be described as

1 $$ \frac{{dx_{i} }}{dt} = x_{i} G\left( {v, {\varvec{u}}, {\varvec{x}}} \right) $$

and the change in the strategy value of species $i$ as

2 $$ \frac{{du_{i} }}{dt} = \sigma_{{u_{i} }} \frac{{\partial G\left( {v, {\varvec{u}}, {\varvec{x}}} \right)}}{\partial v} $$

where both expressions are evaluated at $v = {u}_{i}$. Here, ${\sigma }_{{u}_{i}}$ determines the speed at which the strategy of species $i$ climbs the fitness gradient. These dynamics play out on an adaptive landscape defined as the plot of $G$ versus $v$ for a fixed $({\varvec{u}}, {\varvec{x}})$. If there is negative density dependence $\frac{\partial G}{\partial {x}_{i}}<0\,\forall i$, an increase in ${x}_{i}$ will lower the fitness landscape for all values of $v$ and the current strategy ${\mathbf{u}}$. As a result, changes in ${u}_{i}$ will move in the direction of the fitness gradient.

Under Darwinian dynamics or adaptive dynamics [1], a number of key results emerge. First, there are eco-evolutionary *singular points* where $G(\cdot )=0$ and $\frac{\partial G}{\partial v}=0$ for ${\varvec{u}}$ with positive population sizes ${{\varvec{x}}}^{*}$. These pairs of $({u}_{i}, {x}_{i}^{*})$ occur at minima, maxima or inflection points on the adaptive landscape. Second, the coalition of singular points will form an ESS if the singular points are at global maxima of the adaptive landscape. Third, for the coalition of singular points, we will define the points as *n-*species *convergent stable* if for any ${u}_{i}$ in the coalition, strategies in a close neighborhood of ${u}_{i}$ can be invaded by strategies that are closer to ${u}_{i}$ for each $i$. Fourth, the coalition of singular points will form a *neighborhood invader strategy* (NIS) if the singular points are at minima of the adaptive landscape under entrant strategy variation. Fifth, for the coalition of singular points, we will define the points as *n-*species mutually invadable if for any ${u}_{i}$ in the coalition, two strategies in a sufficiently close neighborhood of ${u}_{i}$, and on opposite sides of the maximum on the fitness landscape can mutually invade each other on an ecological time scale for each $i$. We note that convergence stability is not well defined for higher dimensional singular points [12].

Conditions for the first property of maximum or minimum on the adaptive landscape has been proven for any value of $n$ and for any dimensionality of ${u}_{i}$ [24, 42]. Conditions for convergence stability and NIS have been formalized for cases where there is a single species, $n=1$, and for scalar-valued ${u}_{i}$ [6]. Mutual invasibility around a singular point has only been formalized for scalar-valued ${u}_{i}$ [5, 6].

With these conditions, a number of key results emerge for our present exploration of ESS and non-ESS communities:

1. With scalar-valued strategies, for two species of the *G*-function to coexist, they must be separated by at least one peak or valley of the adaptive landscape.
2. If the coexisting species, at their equilibrium population sizes, are all on global peaks of the adaptive landscape, their fitness, $G(\cdot )$, will be $0$ at such points, the fitness of all other strategies on that landscape will be negative, and this community of species, $({\varvec{u}},{\varvec{x}})$, is an ESS where no alternative species with an infinitesimally small population can invade.
3. For a single species ESS, $n=1$, that is NIS, via mutual invasibility, it is possible to have up to $s+1$ coexisting species as a non-ESS community where $s$ is the dimensionality of ${u}_{i}$.
4. By way of conjecture, for an ESS that is NIS, mutual invasibility makes it possible to have non-ESS communities with up to $n\cdot (s+1)$ coexisting species where clusters of $s+1$ species occur directly in the neighborhood of each ${u}_{i}^{*}$ of $({{\varvec{u}}}^{*},{{\varvec{x}}}^{*})$
5. By way of conjecture, for hypersaturated non-ESS communities of coexisting species, the coexisting species associated with a particular ${u}_{i}^{*}$ will converge on $({u}_{i}^{*},{x}_{i}^{*})$ if each species strategy is allowed to evolve according to the Darwinian dynamics.

In the following, we use *G*-functions that generate Lotka–Volterra competition equations to illustrate and further these conjectures, as well as suggest additional properties of ESS and hyper-diverse non-ESS communities. We consider models of competition with either a scalar-valued strategy along a single niche axis, or a vector-valued strategy based on two niche axes. For the single strategy, we examine how communities with 1, 2, 3, 4 or 5 coexisting species at the ESS can sustain up to 2, 4, 5, 7 and 8 species when the community is not at its ESS (up to or close to twice the number of species in the hypersaturated community relative to the ESS community). For the vector-valued strategy with two quantitative traits we examine how communities with a single-species ESS can sustain up to 3 species in a non-ESS community. While necessary and sufficient conditions for convergence stability, neighborhood invader strategies and mutual invasibility properties for ESSs with multiple species or vector-valued strategies remain elusive, we can use these examples to offer several conjectures that aim to advance evolutionary game theory, niche coevolution with communities of competing species, and species diversity in ESS and non-ESS communities.

### The Model

We consider a *G*-function that generates Lotka–Volterra competition equations:

3 $$ G \left( {v,{\varvec{u}},{\varvec{x}}} \right) = r \left( { \frac{{K\left( v \right) - \mathop \sum \nolimits_{j = 1}^{n} x_{j} \alpha (v,u_{j} )}}{K\left( v \right)}} \right) $$

where carrying capacity $K(v)$ is influenced by the strategy $v$ of the focal individual and the competition coefficient $\alpha (v,{u}_{j})$ depends on the strategy of the focal individual and strategies of others.

*Model for a scalar-valued strategy*: For the case of a scalar-valued strategy, $s=1$, we use the following forms for $K\left(v\right)$ and $\alpha \left(v, {u}_{i}\right):$

4 $$ K\left( v \right) = K_{max} \exp \left( { - \frac{{v^{2} }}{{2\sigma_{K}^{2} }}} \right) $$

where ${K}_{max}$ is the maximum carrying capacity achieved when $v$=0, and carrying capacity declines as a Gaussian function as $v$ deviates in either direction from $v$=0 at a rate determined by ${\sigma }_{K}^{2}$ where a larger value of ${\sigma }_{K}^{2}$ means *K* declines more slowly with $v$ around 0. The carrying capacity curve can be thought of as the resource axis, $v$ as position along that axis, and ${\sigma }_{K}^{2}$ as the breadth of the resource axis. Holding all else equal, a wider resource axis (larger ${\sigma }_{K}^{2}$) permits more species to coexist by equalizing the fitness of differing species, which is a criterion of the ESS, while a narrower resource axis (smaller ${\sigma }_{K}^{2}$) causes fewer species to occur via niche partitioning by introducing sharp fitness differences among similar species [10].

5 $$ \alpha \left( {v,u_{j} } \right) = 1 + \exp \left( { - \frac{{\left( {v - u_{j} + \beta } \right)^{2} }}{{2\sigma_{\alpha }^{2} }}} \right) - \exp \left( { - \frac{{\beta^{2} }}{{2\sigma_{\alpha }^{2} }}} \right) $$

where the Gaussian function implies that like competes most with like, and this competition is somewhat asymmetric. Individuals with larger strategy values have a greater competitive effect on those with smaller values than vice-versa. The degree of this asymmetry is determined by the “bully” term $\beta \ge 0$. When $\beta =0$, competition is symmetrical and becomes increasingly asymmetric as β increases. As expected of Lotka-Volterra models, intraspecific competition yields a competition coefficient $\alpha \left({u}_{i}, {u}_{i}\right)=1$. As the deviation between the competitors’ strategies $\left(v-{u}_{i}\right)$ goes to infinity, the competition coefficient converges on a positive value that increases with $\beta$:

6 $$ \alpha \left( {v, u_{i} } \right) = 1 - \exp \left( { - \frac{{\beta^{2} }}{{2\sigma_{\alpha }^{2} }}} \right) $$

The term ${\sigma }_{\alpha }^{2}$ determines the width of the competitive niche axis. Small values mean that competition between two species declines rapidly as their strategies diverge, and vice-versa for large values. Depending upon the fixed value of ${\sigma }_{K}^{2}$, the number of species at the ESS either declines as ${\sigma }_{\alpha }^{2}$ increases, or at first increases and then declines. Thus, there is an interesting interaction between ${\sigma }_{\alpha }^{2}$ and ${\sigma }_{K}^{2}$, where species diversity at the ESS always increases as ${\sigma }_{K}^{2}$ increases but where the relationship between ${\sigma }_{\alpha }^{2}$ and species diversity at the ESS is not necessarily monotonic (Fig. S3, supplementary material). Other parameters of the model do not influence the number of species at the ESS, and it appears that there is just one ESS for any fixed parameter values [42].

*Model for a vector-valued strategy*: We use a bivariate form of the prior scalar-valued G-function to permit two resource or niche axes:

7 $$ K\left( {\varvec{v}} \right) = K_{max} \exp \left( { - \frac{{v_{1}^{2} + av_{2}^{2} }}{{2\sigma_{k}^{2} }}} \right) $$

8 $$ \alpha \left( {{\varvec{v}},{\varvec{u}}_{j} } \right) = 1 + \exp \left( { - \frac{{\left( {\left( {v_{1} - u_{j1} } \right) + b\left( {v_{2} - u_{j2} } \right) + \beta } \right)^{2} }}{{2\sigma_{\alpha }^{2} }}} \right) - \exp \left( { - \frac{{\beta^{2} }}{{2\sigma_{\alpha }^{2} }}} \right) $$

The strategy of the focal individual now has two components ${\varvec{v}} = ({v}_{1},{v}_{2})$, where each species’ strategy is now a vector ${{\varvec{u}}}_{i} = ({u}_{i1},{u}_{i2})$ and the strategies in the overall community of $n$ species is a vector of vectors: ${\varvec{u}}=({{\varvec{u}}}_{1},{{\varvec{u}}}_{2},\dots {{\varvec{u}}}_{n})$. Carrying capacity, $K({\varvec{v}})$, is now a bivariate Gaussian that reaches a maximum of ${K}_{max}$ at ${\varvec{v}} = (\text{0,0})$ and the term $a$ allows for the rate of decline in $K$ with ${v}_{2}$ to be larger ($a>1$) or smaller ($0<a<1$) than that of ${v}_{1}$. Similarly, the competition coefficient is bivariate, preserving the property that $\alpha ({{\varvec{u}}}_{i},{{\varvec{u}}}_{i})=1$. The bully term $\beta$ remains the same, and the parameter $b$ allows the rate of decline in $\alpha$ with ${v}_{2}$ to be larger ($b>1$) or smaller ($0<b<1$) than that of ${v}_{1}$. Table 1 shows the default values of the parameters that were used for all the simulations and results presented in this study, unless otherwise mentioned.

**Table 1:** Default parameter values used in this study

$r$	${K}_{max}$	${\sigma }_{\alpha }^{2}$	$\beta$	${\sigma }_{{u}_{i}}$	$a$	$b$	${\sigma }_{K}^{2}$
0.25	100	4	2	0.5	0.9	1.1	Varied

*Approach to non-ESS simulations*: At an ESS, there is ecological stability where $G(\cdot )=0$ and evolutionary stability where $\frac{\partial G}{\partial v}$ $=0$, and at evolutionary equilibrium, all strategies reside on global, equal fitness peaks, forming a *saturated* ESS community. However, if evolutionary dynamics are slower than ecological dynamics, and if natural systems have fluctuating and variable selection pressures in ecological time, then we envision non-ESS communities to be those where communities approach an ecological equilibrium, but not an evolutionary equilibrium. Thus, to analyse non-ESS communities, our approach was to turn off evolutionary dynamics and only permit ecological dynamics. We sought stable non-ESS communities in the following way:

(1) Identifying ESS communities $({{\varvec{u}}}^{*},{{\varvec{x}}}^{*})$ where $G(\cdot )=0$: We seeded $p$ populations with initial trait values ${{\varvec{u}}}^{0}$ uniformly spaced in the interval [-10, 10], and initial population sizes ${{\varvec{x}}}^{0}$ randomly chosen from the interval [$\frac{{K}_{max}}{125},\frac{{K}_{max}}{100}$] where ${K}_{max}=100$ is the maximum carrying capacity. $p$ was set to 10 unless otherwise mentioned. The system was then allowed to reach both ecological and evolutionary equilibria following the dynamics given by Eq. 1 and Eq. 2. For our numerical solutions, ecological equilibrium and evolutionary equilibrium are said to be reached at a time $t$ when both the change in frequencies of the populations and change in trait values ${\varvec{u}}$ of the populations are less than ${10}^{-7}$ for 5000 consecutive simulated time units respectively (Eqs. 9 and 10).

9 $$ \left| {\frac{{x_{i}^{\tau } }}{{\mathop \sum \nolimits_{i} x_{i}^{\tau } }} - \frac{{x_{i}^{{\tau - {\Delta }t{ }}} }}{{\mathop \sum \nolimits_{i} x_{i}^{{\tau - {\Delta }t}} }}} \right| \le 10^{ - 7}\,\forall i \in \left\{ {1,..,p} \right\}, \forall \tau \in \left[ {t - 5000, t} \right] $$

10 $$ \left| {u_{i}^{\tau } - u_{i}^{{\tau - {\Delta }t}} } \right| \le 10^{ - 7}\, \forall i \in \left\{ {1,..,p} \right\}, \forall \tau \in \left[ {t - 5000, t} \right] $$

The trait values possessed by the populations at equilibrium are assumed to be the ESS trait values ${{\varvec{u}}}^*$. The population sizes ${{\varvec{x}}}^*$ at equilibrium are assumed to be the population sizes of the ESS trait values. In case multiple populations evolve to have the same trait value at equilibrium, their corresponding populations sizes are summed and are considered to be the same species. Thus, ${{\varvec{u}}}^{*}$ has elements ${u}_{k}^{*}$ where ${u}_{k}^{*}$ is the $k$th ESS trait value, $k \in \left\{1,\dots , {n}_{ESS}\right\}$, where ${n}_{ESS}$ is the number of unique trait values possessed by species at ESS.

(2) We open an invasion window, an interval around each ESS defined by positive fitness $$({U}^{+}= \left\{v\in {U}_{N}|G\left(v, {\varvec{u}}, {\varvec{x}}\right)\ge 0\right\}, \text{where }{U}_{N}=\left\{v\right| \left|v-{u}_{i}^{*}\right|\le 0.25\}$$ is the set of trait values in the neighbourhood of an ESS trait $${u}_{i}^{*})$$, by moving one or more $${u}_{i}$$ values off of its peak by a value $$\delta \in \{0.01, 0.05, 0.1, 0.2, 0.3\}$$. Similarly, we add a second species on the other side of that peak $$(\delta \in \{-0.3, -0.2, -0.1, -0.05, -0.01\})$$, within the invasion window.

(3) We then allow only the ecological dynamics (Eq. 1) to evolve with this new over-saturated community and keep ${\varvec{u}}$ fixed. (4) The resulting non-ESS community was considered ecologically stable once the populations converged to equilibrium according to the condition given by Eq. 9. (5) If a population went extinct, we tried the following strategies to find a new set of trait values for the members of the non-ESS community, in an attempt to maximize the number of surviving, coexisting species:

- Change its trait value by $\epsilon$, where $\epsilon$ is randomly chosen from the interval $\left[0.01, 0.1\right]$ in the direction of the invasion window formed around its peak without crossing the ESS value
- Change the trait value of its counterpart (on the other side of the peak) by $\epsilon$, where $\epsilon$ is randomly chosen from the interval $\left[0.01, 0.1\right]$, away from the invasion window formed around its peak
- Change the trait value of its neighbours at higher trait values, if any, by $\epsilon$, where $\epsilon$ is randomly chosen from the interval $\left[0.01, 0.1\right]$, in the direction of increasing trait value
- Change the trait value of its neighbours at lower trait values, if any, by $\epsilon$, where $\epsilon$ is randomly chosen from the interval $\left[0.01, 0.1\right]$, in the direction of decreasing trait value

We repeated step (5) at least 150 times to find a non-ESS community with twice the number of species as ESSes in the 1, 2, 3, 4, and 5-species ESS cases.

It is important to note that if the evolutionary dynamics were allowed to evolve as well (${\varvec{u}}$ is not fixed), such communities would not be evolutionarily stable, and one species would displace the other to reach the peak of the fitness landscape. However, if evolutionary dynamics are slower than ecological dynamics, such communities could persist for a long period of time in nature.

## Results

*Scalar-valued strategy*: It is well established that ${\sigma }_{K}^{2}$ is a bifurcation parameter determining the number of species at the ESS [18, 21, 31]. If $\beta \ne 0$, then as ${\sigma }_{K}^{2}$ increases, the niche breadth increases, and the ESS increases successively from 1, 2, 3, … to *n* species. If $\beta =0$ (competition is symmetric), then the system jumps from a single species ESS to one with an infinite number of species as ${\sigma }_{K}^{2}$ increases [15].

**Fig. 1:**
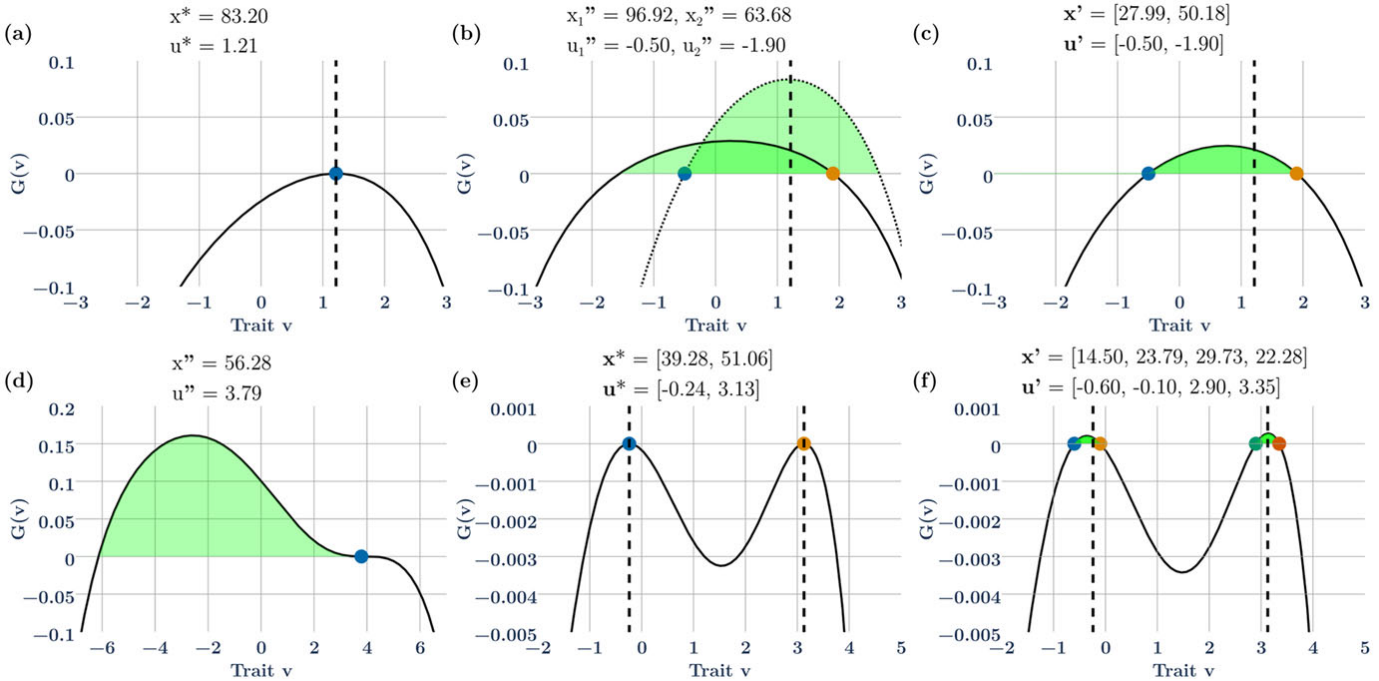
Results for the single strategy (${\upsigma }_{\text{K}}^{2}=4$) (a, b, c) and two strategy (${\upsigma }_{\text{K}}^{2}=12.5$) (d, e, f) ESS cases. Colored circles represent species’ strategies. Solid and dotted lines represent the value of the G-function vs strategy value when the resident has the strategy marked by the respective colored circle on the lines. The G-function lines of all the species overlap in c, d, e, and f, and only one line is visible. The dashed vertical lines show the location of the ESS strategy values on the x axis. (**a**) Single strategy ESS. (**b**) Invasion windows (light green shaded regions) of each of the two non-ESS species with strategies above and below the ESS when they are the sole population and are at ecological equlibrium. The overlap of the respective invasion windows is the bright green region. (**c**) Adaptive landscape when the two non-ESS species coexist. (**d**) Convergent stable minimum before reaching two strategy ESS. (**e**) Two strategy ESS. (**f**) Adaptive landscape when 4 non-ESS species coexist

*Single species ESS*: When we have a single species, the ESS (e.g., $\sigma_{K}^{2} = 4$) is NIS and exhibits mutual invasibility for diverse pairs of ($u_{1}, u_{2}$) (Fig. 1a–c). For the example in Fig. 1a, the ESS strategy, $u^{*} = 1.21$, is greater than the value that would maximize carrying capacity ($u=0$). Thus, ${x}^{*} = 83.2$ is less than the potential of ${K}_{max}=100$ at $u=0$, illustrating that under frequency-dependent selection, natural selection does not necessarily maximize equilibrium population size. At the ESS, no other species can exist within this community – it is both ecologically and evolutionarily stable. As a hypersaturated, non-ESS community, the two species with strategies ${\varvec{u}}^{\prime } = \left( { - 0.50, \,1.90} \right)$ and equilibrium population sizes of $x^{\prime } = (27.99,50.18)$ can coexist. It should be noted that this successful coexistence is not limited to these specific values of strategies. In our simulations, coexistence was easily achieved for numerous strategy pairs as long as they straddled the ESS. This can be seen in Fig. 1b, by each strategies’ invasion window. The invasion window of strategy *i* is defined as the set of strategies ${U}_{i}$ such that $G(v,{u}_{i},{x}_{i}^{*}) > 0$ for all $v \in {U}_{i}$. In this example, mutual invasibility is always possible, and indeed well characterized since ${u}_{1}$ and ${u}_{2}$ are elements of ${U}_{2}$ and ${U}_{1}$, respectively. With a single species ESS with scalar valued strategies, the hypersaturated non-ESS community can have up to 2 coexisting species (Fig. 1c). If evolution occurs, natural section will drive both species’ strategies to the ESS (Fig. 1a). Note that there are an infinite number of strategy pairs that can coexist. But, the range of these strategy pairs are constrained to satisfy mutual invasibility.

*Two species ESS*: With a broader niche breadth (${\sigma }_{K}^{2}$ = 12.5) the ESS contains two species ${\varvec{u}}^{*} = \left( { - 0.24, 3.13} \right)$ at ${\varvec{x}}^{*} = \left( {39.28, 51.06} \right)$ (Fig. 1e). If the community begins with a single species, its strategy will evolve to a convergent stable minimum of ${u}=3.79$ at $x=56.28$ (Fig. 1d). This under-saturated community will remain at this minimum unless another species with a different strategy is introduced. With two species, their strategies will evolve to the ESS (Fig. 1e). If one or the other of these species is removed from the community, then the other species increases in abundance and possesses an invasion window that includes the other strategy of the ESS (Fig. 1d, for example).

**Fig. 2:**
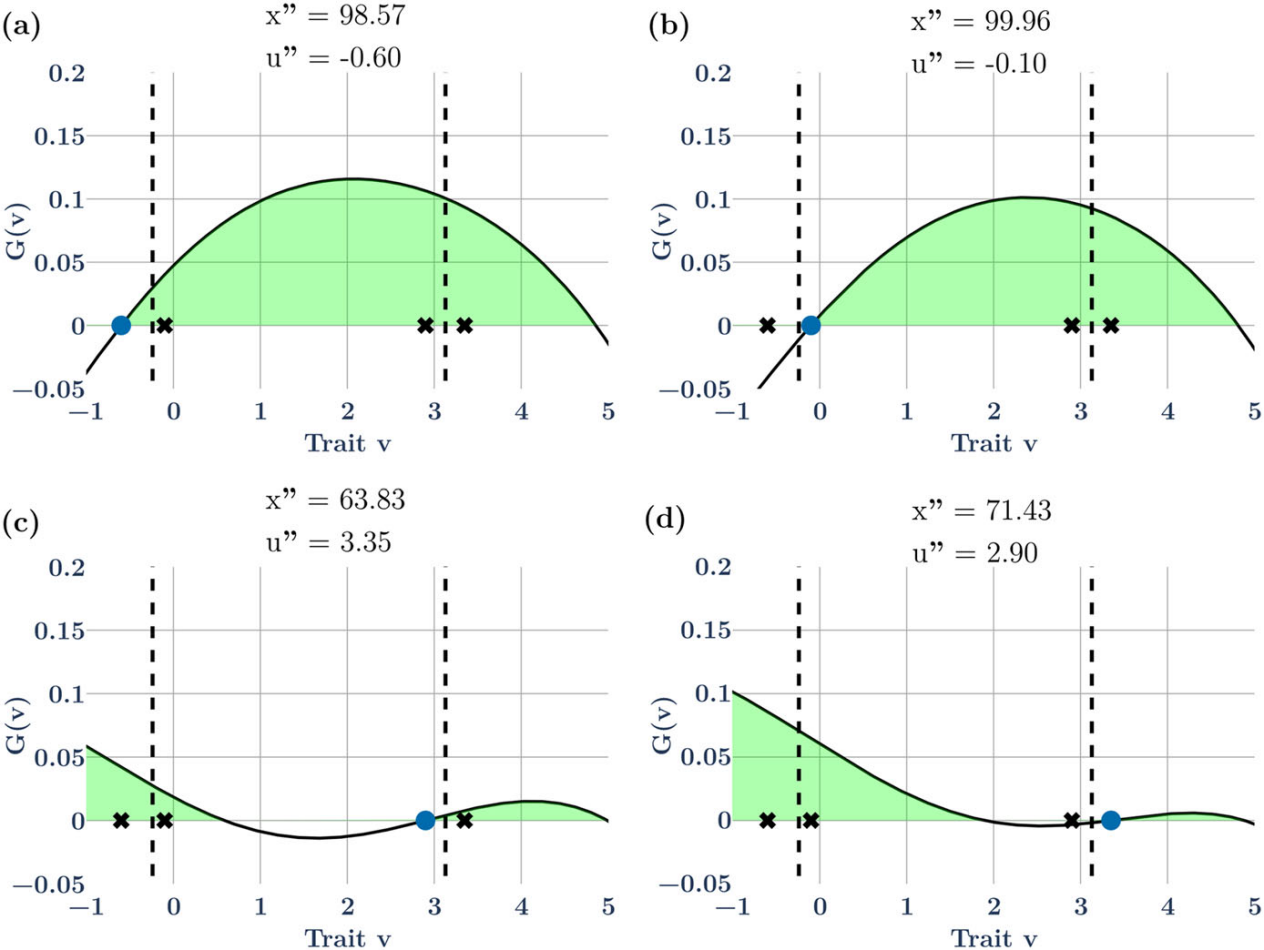
The non-ESS community shown in Fig. 1f has strategies $\mathbf{u}=(-0.60, -0.10, 2.90, 3.35)$. Here, the invasion windows of each of the 4 non-ESS species in Fig. 1-f are shown as if the starting conditions reflected each of those four species alone. Invasion windows (light green shaded regions) of species 1 (a) and 4 (d) contain all the other species’ strategies. Invasion windows of species 2 (b) and 3 (c) do not contain one of the other three species’ strategies. Species 3 (c) and 4 (d) have discontinuous invasion windows with valleys separating the two regions of the invasion window. Colored circles represent species’ strategies. Solid lines represent the value of the G-function vs strategy value when the resident has the strategy marked by the respective colored circle on the lines. The vertical dashed lines show the location of the ESS strategy values on the x axis

**Fig. 3:**
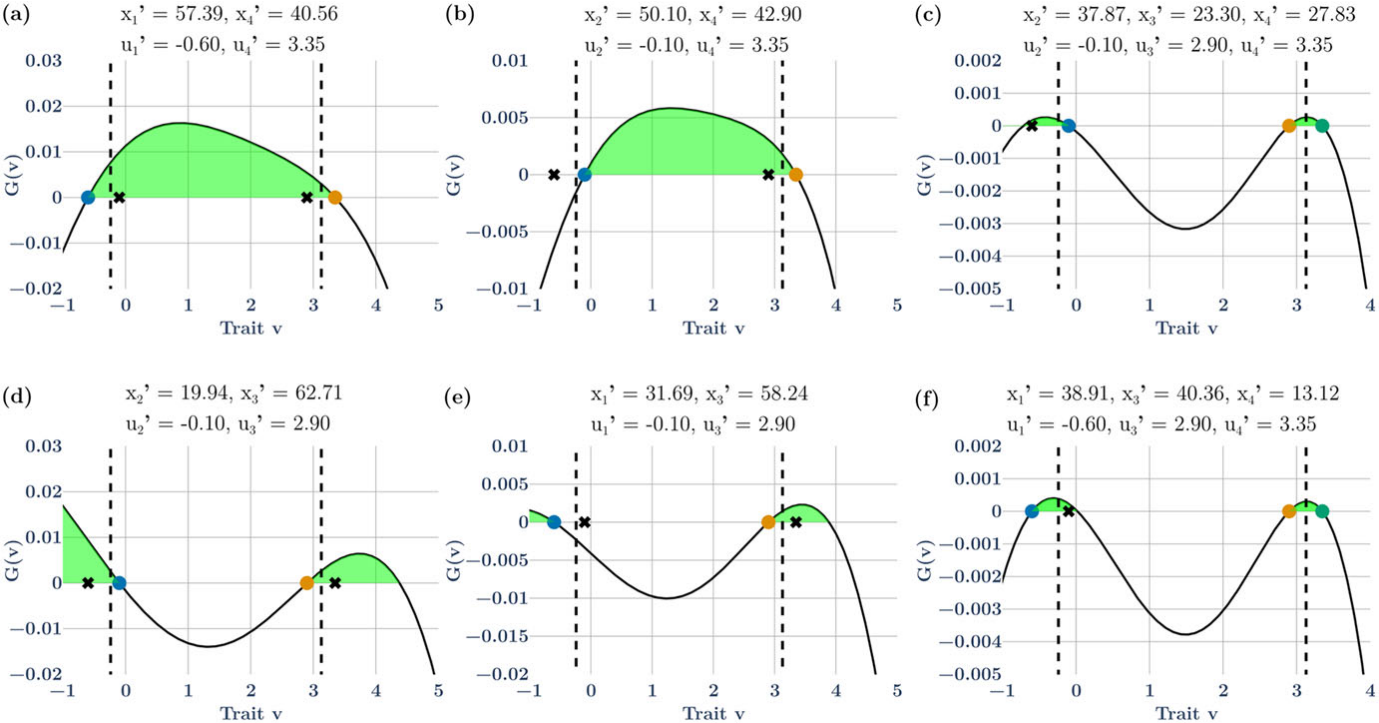
The non-ESS community shown in Fig. 1f has strategies $\mathbf{u}=(-0.60, -0.10, 2.90, 3.35)$. Here, the invasion windows are shown for all combinations of two species as the initial conditions (**a**, **b**, **d**, **e**) and two three species initial conditions (**c**, **f**) non-ESS species coexist. Invasion windows when species 1 and 4 coexist (a), 2 and 3 coexist (d) contain all other strategies within them, whereas 2 and 4 (b) and 1 and 3 (e) do not. The two possible 3-species combinations have invasion windows that contain the fourth strategy (c, f). Colored circles represent species’ strategies. The solid line represents the value of the G-function vs strategy value when the coexisting residents have the strategies marked by the colored circle on the lines. The vertical dashed lines show the location of the ESS strategy values on the x axis

For this example, there exist combinations of 4 species that can coexist as pairs of species straddling the strategies of the ESS. Here we explore one such community ${\varvec{u}}^{\prime } = \left( { - 0.60, - 0.10, 2.90, 3.35} \right)$ at ${\varvec{x}}^{\prime } = \left( {14.50, 23.79, 29.73, 22.28} \right)$ (Fig. 1f). Because there are 2 possible invasion windows for each ESS (e.g., Fig. 1b), when considering all possible subsets of the 4 species, there are now 14 possible invasion windows for a hypersaturated community built from the two-ESS example: 4 with 1 species (Fig. 2), 6 with 2 species (Fig. 3a, b, e, f), and 4 with 3 species (Fig. 3c, d). Not all of these invasion windows are continuous sets, and not all of them can occur due to competitive exclusion within particular combinations of species. In the next section we describe these subsets of invasion windows to show: (i) that unlike a 1-species ESS where it is immediately possible to determine mutual invasibility, when there is more than 1 species it becomes difficult to determine mutual invasibility for subsets of the hypersaturated community because these subsets may not contain the strategy values of other species; (ii) that the ESS might not be in the invasion window of the subset of species; (iii) that the invasion window may not be continuous, and (iv) that not all subsets will allow successful invasion of all species.

First, consider, as shown in Fig. 2a, the invasion window for species 1 (blue dot) with respect to the other three species (stars) shown in Fig. 1f. Here, all of the other three species reside in the invasion window of species 1 (Fig. 2a; $u_{2}^{\prime } , u_{3}^{\prime }\,\text{and}\,u_{4}^{\prime } { } \in U_{1}^{\prime }$). If this world with species 1 was invaded by the other 3 species, then the invasion window shown in Fig. 2a could lead to a 4-species hypersaturated community because it contains the invasion window of the other three species, it contains the ESS, and the window is continuous. However, this situation is not always true for all possible starting conditions. For example, both species 1, $u_{1}^{\prime },$ and the first strategy of the ESS, ${u}_{1}^{*},$ are absent from the invasion window of non-ESS species 2, $u_{2}^{\prime }$ (Fig. 2b), meaning a world starting with $u_{2}^{\prime }$ would lead to a 3-species hypersaturated community not the 4-species hypersaturated community shown in Fig. 1f. The invasion window of species 3, $u_{3}^{\prime }$, contains all of the other species (Fig. 2c), whereas non-ESS species 3, $u_{3}^{\prime }$_,_ and the second ESS species, $u_{2}^{*}$, are absent from non-ESS species 4’s invasion window, $u_{3}^{\prime }$ (Fig. 2d).

Second, consider the non-ESS species from Fig. 1f in 2 species combinations (Fig. 3a, b, d, e). Of the 6 possible combinations of 2 species out of a pool of 4 species, two combinations cannot occur because species 1 and 2 can never coexist as a two species pair (Fig. 2b; species 2 outcompetes species 1), and species 3 and 4 can never coexist as a two species pair (Fig. 2d; species 4 outcompetes species 3). However, the remaining 4 of 6 2-species invasion windows can be drawn. For species 1 and 3, species 2, $u_{2}^{\prime }$, and the first ESS species, $u_{1}^{*}$, are not in the pair’s invasion window, $u_{1,3}^{\prime }$ (Fig. 3e). For species 1 and 4, species 2 and 3, and both ESS species are within the pair’s invasion window, $u_{2,4}^{\prime }$ (Fig. 3a). The same holds for the invasion window of species 2 and 3, $u_{2,3}^{\prime }$ (Fig. 2d). The invasion window of species 2 and 4, $u_{2,4}^{\prime }$, excludes species 1, $u_{1}^{\prime }$ (Fig. 2b). In summary, only 4 of the possible six 2-species combinations are possible and have invasion windows. This highlights how worlds with different starting conditions might lead to different non-ESS communities and depends on the invading species involved. It also shows how invasion windows are not necessarily continuous when there is a valley between peaks on the adaptive landscape.

Lastly, there are 4 possible combinations of the four non-ESS species in 3-species combinations but as above, not all of them can occur due to competitive exclusion within particular combinations. Two 3-species combinations are not possible: species 1, 2, and 3; and species 1, 2, and 4 cannot coexist and thus there are no invasion windows for these combinations. The invasion window of species 1,3, and 4, $u_{1,2,4}^{\prime }$, contains species 2 and both species of the ESSs (Fig. 3f). The invasion window of species 2, 3, and 4, $u_{2,3,4}^{\prime }$, contains species 1 and both species of the ESSs (Fig. 3c). Thus, of the 14 possible initial conditions leading to a 4-species hypersaturated community through invasions, only 6 (Fig. 2a, c; Fig. 3a, c, d, f) could lead to the fully hypersaturated 4-species non-ESS community shown in Fig. 1f because 4 cannot occur due to competitive exclusion and 4 of them lead to fewer than 4 coexisting species in the final hypersaturated non-ESS community (Fig. 2b, d; Fig. 3b, e). Furthermore, 6 of these possible invasion windows are discontinuous while only 4 are continuous.

**Fig. 4:**
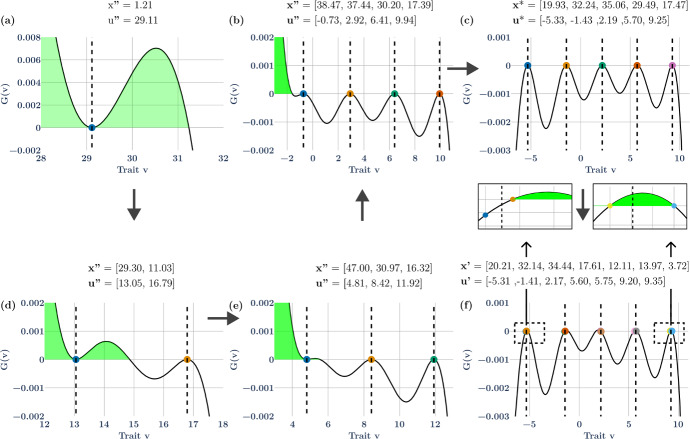
**a**, **d**, **e**, **b** show stages of evolutionary branching with convergent stable equilibria before reaching the 5 species ESS (**c**). Adding species on each side of the 5 ESSs leads to 3 species going extinct and 7 species coexisting (**f**). The insets above (f) show the two species around peak 1 (left inset, blue species does not survive) and peak 5 (right inset, both species survive). Colored circles represent species’ strategies. The solid line represents the value of the G-function vs strategy value when the residents have the strategies marked by the colored circle on the lines. The vertical dashed lines show the location of the ESS strategy values on the x axis. The (bright-) light-green shaded regions represent (overlapping) invasion windows (Color figure online)

*Five species ESS*: With a larger ${\sigma }_{K}^{2}$, the ESS transitions from 3 to 4 to 5 species (Fig. 4 a, b, d, e), and more due to speciation. Here, we consider ${\sigma }_{K}^{2}$ = 96 which results in a five species ESS $\mathbf{u}^* = ( - 5.33, - 1.43,2.19,5.70,9.25),$ ${\varvec{x}}^{*} = \left( {19.93, 32.24, 35.06, 29.49, 17.47} \right)$ (Fig. 4c). Consider the undersaturated communities. With a single species, Darwinian Dynamics leads to a convergent stable minimum ($u^{\prime \prime } = 29.11,\,x^{\prime \prime } = 1.21$; Fig. 4a). With speciation from even a tiny mutation, or the addition of another species, the eco-evolutionary dynamics result in a convergent stable singular point with the higher valued strategy, $\left( {u^{\prime \prime } = 16.79,\,x^{\prime \prime } = 11.03} \right)$ at a peak and the lower valued, $\left( {u^{\prime \prime } = 13.05,\,x^{\prime \prime } = 29.30} \right)$, at a minimum (Fig. 4d). Similarly, the 3-species convergent stable equilibrium, ${\varvec{u}}^{\prime \prime } = \left( {4.81, 8.42, 11.92} \right)$ and ${\varvec{x}}^{\prime \prime } = \left( {47.00, 30.97, 16.32} \right)$, has the two higher strategies at peaks and the lowest strategy at a minimum (Fig. 4e). At four species, ${\varvec{u}}^{\prime \prime } = \left( { - 0.73, 2.92, 6.41, 9.94} \right)$ and ${\varvec{x}}^{\prime \prime } = \left( {38.47, 37.44, 30.20, 17.39} \right)$ all converge to peaks of the adaptive landscape, yet are not at an ESS, there is an invasion window at lower values, but a valley separates the lowest extant species from the invasion window (Fig. 4b). The 5-species ESS can only be established by adding a species with a strategy value far from the lowest value in the 4-species equilibrium (Fig. 4c).

**Fig. 5:**
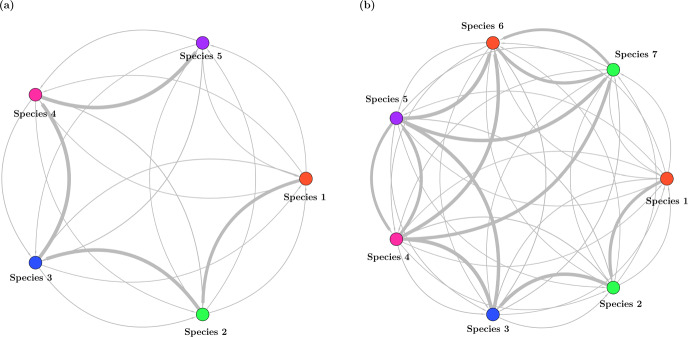
Network depicting the effect of competition of each species on the others for the 5 ESS species (a) and 7 non-ESS species (b) systems. The nodes represent the species and edge thickness denotes the effect of competition of the source species on the sink species. In (a), the effect of competition is greatest from the immediate neighbor with higher strategy value. In hypersaturated communities, the interactions are stronger because more species are very similar (b)

The 5-species ESS results in a matrix of interaction coefficients showing asymmetries as species with higher strategy values have a stronger effect on species with lower strategy values than vice-versa (Fig. 5a). Each species is most affected by its next nearest neighbor with a higher strategy value because the bully term intensifies this effect for larger strategy values. An ecologist studying nature would see this 5-species community without necessarily knowing the underlying adaptive landscape connecting them. Removal experiments are often used by experimental biologists to give insights into species interactions, and we undertake this type of analysis to explore the non-ESS community.

Removing species 5 (the species that sacrifices carrying capacity for competitive dominance) results in the extinction of species 1 (the species that sacrifices carrying capacity to avoid competition). This comes about through diffuse competition. Removal of species 5 releases species 4 from competition, depressing species 3, releasing species 2 that then drives species 1 extinct. Species 5 would be seen as a keystone species.

Removing species 4 results in a shorter but similar cascade of lowered and heightened competition as species 2 now goes extinct. Species 3 removal, like removing species 5, drives species 1 extinct, while removals of species 1 or 2 result in no additional species losses. Species 3 and 4 are keystone, species 1 and 2 not.

At first, we expected to achieve a hypersaturated community of 10 species by locating 5 pairs of species around each peak of the ESS. Each species pair would have strategies very close together (we tried differences of 0.05, 0.1, 0.2, and 0.3 around the ESS peak between potentially similar coexisting species), and each pair straddled an ESS value. We were unsuccessful. Upon viewing the shifts in the adaptive landscape resulting from the collapse of these 10 species communities, we attempted to shift the pair rightwards or leftwards depending upon the gradient (detailed explanation can be found in the methods section). This also failed to produce communities with 10 coexisting species.

What did we learn from 150 attempts? We regularly achieved hypersaturated communities of 7 species (see Fig. 4f for an example), and with much trial and error, a community with 8 coexisting species, but never one with 9 or 10. We make the following observations regarding hypersaturated communities: (1) they had some pairs of species associated with peaks of the ESS, (2) coexisting pairs did not always straddle the ESS of their peak, (3) obtaining pairs for the higher ESS peaks appeared easier than the lower ESS peaks, (4) we were never able to obtain a coexisting pair for the 2nd – 5th peaks of the ESS when all other species were fixed at their ESS values, and (5) we were never able to obtain a coexisting pair of species around the 1st peak of the ESS even when all other species were fixed at their ESS.

After 150 attempts to achieve hypersaturated non-ESS communities, we make the following conjectures without proofs. First, considering each peak of the ESS, in turn, the 1st peak is not locally NIS, while the remaining 4 peaks are locally NIS. We observe a similar behavior in the 3-species and 4-species ESS cases too (Fig S1, supplementary material). Second, the lack of NIS of the 1st peak precludes a 10-species hypersaturated community. Third, the lack of NIS for the first peak may be related to the 4-species undersaturated community having all four species at local maxima of the adaptive landscape. Fourth, the NIS property of the remaining four peaks means that it is possible to have a 9 species community with pairs associated with each of these 4 peaks and 1 species associated with the first – though after 150 attempts, we never actually found a 9-species community. Fifth, the increasing difficulty of finding hypersaturated communities of 7 and then 8 species results from increasing diffuse competition (Fig. 5).

As a final exercise, we can take the 7 species, hypersaturated community shown in Fig. 4f and examine its structure by performing the 7 separate single species removals to see the resulting changes in the remaining 6 species. Figure 5b shows the network of interaction strengths that emerge from these removals. Recall species 1, 2 and 3 of this community reside near ESS peaks 1–3, species 4 and 5 straddle the 4th ESS peak, and species 6 and 7 straddle the 5th ESS peak.

In the 7 species hypersaturated community, removing species 7 (the one with highest strategy value) results in species 5 going extinct, and consequently species 4 and 6 experiencing competitive release. Removing species 6 causes extinction of species 4 and release of species 5 and 7. The removal of species 5 leads to the release of species 4 and 7, and the removal of species 4 causes species 7 to go extinct and the release of species 5 and 6.

So far, species 1, 2, and 3 remain unaffected. However, removal of species 3 causes species 1 and 5 to go extinct while releasing the others. Removing species 2 releases species 1 and leads to extinction of species 7, and removal of species 1 leads to extinction of species 5 and 6 while releasing species 4 and 7. With this hypersaturated community, we see that the effect of competition now goes in both directions (towards higher and lower strategy values) while it only goes towards the lower strategy values in the 5 species ESS community (Fig. 5).

We also considered ESSs with 3 and 4 species (${\sigma }_{K}^{2}$ = 30 and 40, respectively). While it may be possible to get 6 and 8 coexisting species, respectively, in the hypersaturated, non-ESS communities, we were unable to find any. Instead, it was easy to find up to 5 and 7 species, respectively, in the non-ESS communities (see Fig. S2, supplemental material).

**Fig. 6:**
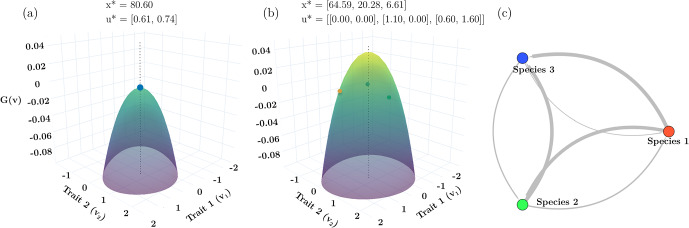
**a** The single species ESS when vector-valued traits are considered. **b** Coexistence of three non-ESS species. **c** Interaction network of the three non-ESS species. Here ${\upsigma }_{\text{K}}^{2}=2$. Colored circles represent species’ (vector-valued) strategies. The surface represents the value of the G-function vs strategy values when the residents have the strategies marked by the colored circle on the surfaces. The vertical dashed lines show the location of the ESS strategy values on the trait 1-trait 2 plane. The color gradient on the surface scale the value of the highest fitness (green) relative to the lowest (purple) invasion windows (Color figure online)

*Single species ESS with vector-valued strategy*: We use the bivariate model of competition with two niche axes to further explore non-ESS communities. A species’ strategy is now a vector with two components, each describing its niche position along one of the axes. As above, for small values of ${\sigma }_{K}^{2}$ the resulting ESS has a single species residing at a global maximum on the hilltop of a 2-D adaptive landscape (Fig. 6a). For our example, we have the single strategy $\mathbf{u}^*=(0.61, 0.74)$ with ${x}^{*} = 80.6$. Note that $\mathbf{u}=(0,0)$ would maximize total population size, 100. But, this “team optimum” is not ESS, and subject to invasion because of the bully term.

**Fig. 7:**
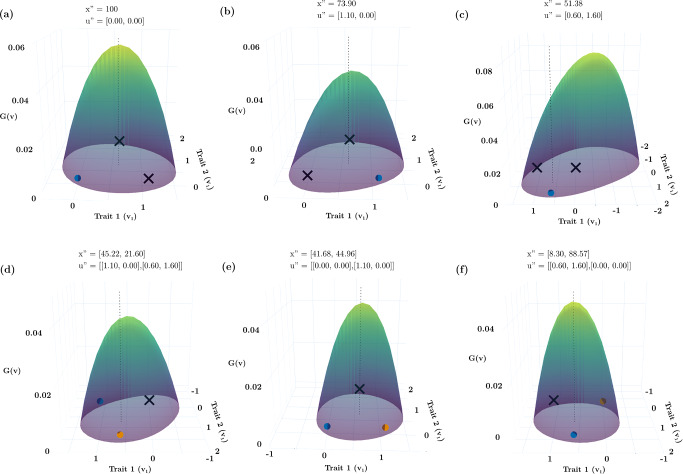
Invasion windows of each of the three non-ESS species (**a**–**c**) and when they coexist in pairs (**d**–**f**) Each invasion window contains the other species’ strategies (marked by crosses). Colored circles represent species’ (vector-valued) strategies. The surface represents the value of the G-function vs strategy values when the residents have the strategies marked by the colored circle on the surfaces. The vertical dashed lines show the location of the ESS strategy values on the trait 1-trait 2 plane. The color gradient on the surface scale the value of the highest fitness (green) relative to the lowest (purple) invasion windows (Color figure online)

As predicted by Durinx et al. [18], it is possible to have a hypersaturated community of 3 coexisting species straddling the single species ESS when the traits are bivariate (Fig. 6b). An example includes ${\varvec{u}}^{\prime } = \left( {\left( {0.00,0.00} \right),\left( {1.10,0.00} \right),\left( {0.60. 1.60} \right)} \right)$ at ${\varvec{x}}^{\prime } = \left( {64.59, 20.28, 6.61} \right)$. Note how the total population size of this community is larger than that of the ESS while smaller than 100. The 3-species hypersaturated community generates 6 invasion windows: 3 single species (Fig. 7a–c) and 3 combinations of 2 species (Fig. 7d–f). In all cases, each invasion window includes the ESS and the other species. Thus, all pairs of species can form stable communities (Fig. 6c).

**Fig. 8:**
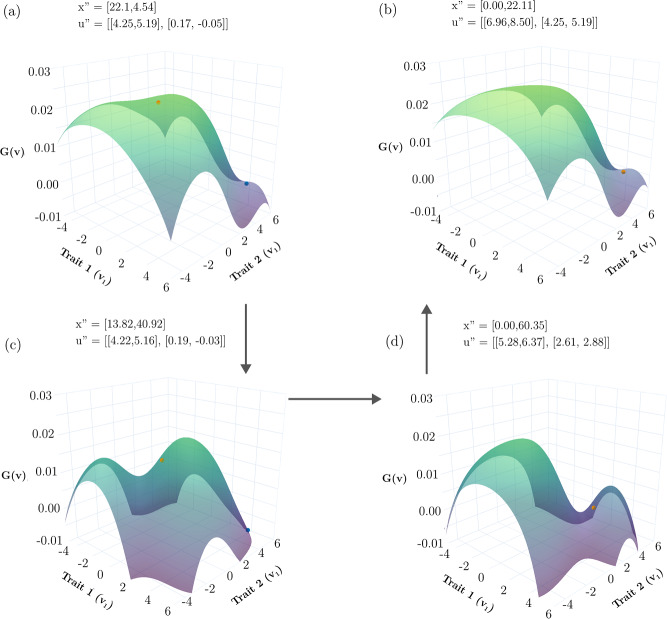
Fitness landscape at different times (progressing anti-clockwise, a-c-d-e) when a new species is added with ${\upsigma }_{\text{K}}^{2}=14$. When a second species is introduced at $\mathbf{u}=(0.17, -0.05)$ (orange dot, top of the landscape in (**a**)) with the resident species at the saddle point (blue dot in (a)), the adaptive landscape changes drastically. Soon, a valley forms (**c**) and the resident is pushed out and goes extinct, and the new species evolves back towards the saddle point (**d**, **b**). Colored circles represent species’ (vector-valued) strategies. The surface represents the value of the G-function vs strategy values when the residents have the strategies marked by the colored circle on the surfaces. The color gradient on the surface scale the value of the highest fitness (green) relative to the lowest (purple) invasion windows (Color figure online)

*Absence of single species ESS with a vector-valued strategy*: We had expected that increasing ${\sigma }_{K}^{2}$ would, as in the case of the scalar-valued strategy, yield ESS communities with successively more species. This was not the case. By increasing ${\sigma }_{K}^{2}$ from 2 to 50 (while keeping ${\sigma }_{\alpha }^{2}=4,\upbeta =2$), the single species ESS did not break down. With a single species, the Darwinian dynamics came to rest at a convergent stable saddle point (similar to Fig. 8b, but not shown) that exhibited a large invasion window indicating a non-ESS community of 1 species. For all attempts to introduce a second species, the resulting transient dynamics resulted in the extinction of the first species as the second species evolved to occupy its place at the saddle point (Fig. 8 provides snapshots of this process). All attempts to introduce up to 11 novel species with strategies sometimes far from the saddle point resulted in Darwinian dynamics leading to a single species at the convergent stable saddle point. At present we have no explanation for this intriguing phenomenon.

## Discussion

Here, we explored non-ESS communities that had ecological dynamics but not evolutionary dynamics, and were both undersaturated and hypersaturated with respect to the number of species in the ESS community had the evolutionary dynamics been turned on. Communities with fewer (undersaturated) or greater (hypersaturated) numbers of coexisting species, though ecologically stable, have invasion windows. In the presence of evolutionary dynamics, this means the extant species will speciate, evolve, or the non-ESS community can be invaded by species with alternative strategies. Undersaturated communities may fill out the ESS community via evolutionary branching where the strategy of one or several of the species evolve to convergent stable minima of the adaptive landscape; or the species’ strategies may evolve to local peaks of the adaptive landscape, where reaching the invasion window requires the introduction of one or more species on the other side of a valley in the adaptive landscape. In this latter case, finding and achieving the ESS community may be challenging via Darwinian dynamics. As our example with a 5-species ESS, simply adding a 5th or 6th species to the community of 4 species residing on local peaks often resulted in transient dynamics that returned the community to the 4-species undersaturated community. In natural evolutionary systems, crossing valleys in the adaptive landscape and achieving the ESS may not be as easy as adding a species on the other side of the fitness valley. In simulated communities, however, applying the ESS maximum principle and numerically solving for the conditions for joint ecological and evolutionary stability (in this case 10 simultaneous equations) offers a simpler path to the 5 species ESS.

Hypersaturated communities involve pairs (scalar-valued strategies; Figs. 1, 2, 3, 4, 5) or triplets (vector-valued strategy with 2 components; Figs. 6, 7, 8) of coexisting species exhibiting mutual invasibility. Each species must reside within the invasion window of another species or some combination of other species. When the ESS community possesses a single species, then these conditions follow directly from proofs provided by Durinx et al. [18]. When the ESS community possesses several strategies, conditions for mutual invasibility remain to be proved. But four conjectures stand out: (1) the maximum number of species in a hypersaturated community is *n*·(*s* + 1) where *n* is the number of species at the ESS and *s* is the dimension of an individual’s strategy; (2) the coexisting species represent pairs (scalar valued strategy), triplets (2-D strartegy), etc. grouped around separate peaks of the adaptive landscape that are near the ESS; (3) the invasion window of one or several species may be discontinuous; (4) a peak of the ESS must be locally NIS for it to possibly support more than one species within the hypersaturated community.

Apaloo [4] derived conditions for finding local NIS conditions in multi-species, scalar-valued/vector-valued (co-)evolutionary games. In an *n* species evolutionary game, the coalition of singular points ${{\varvec{u}}}^{*}=({{\varvec{u}}}_{1}^{*}, {{\varvec{u}}}_{2}^{*}, \dots , {{\varvec{u}}}_{n}^{*})$, which is a vector of vectors, is *NIS* if $G({{\varvec{u}}}_{i}^{*}, {{\varvec{u}}}^{*,i},\boldsymbol{ }{\varvec{x}}({{\varvec{u}}}^{*,i}))>0$ where ${{\varvec{u}}}^{*,i} =(\boldsymbol{ }{{\varvec{u}}}_{1}^{*}, {{\varvec{u}}}_{2}^{*}, \dots , {{\varvec{u}}}_{i-1}^{*}, {{\varvec{u}}}_{i}, {{\varvec{u}}}_{i+1}^{*}, \dots , {{\varvec{u}}}_{n}^{*})$ for any ${{\varvec{u}}}_{i}$ in a close neighborhood of, and distinct from, ${{\varvec{u}}}_{i}^{*}$ for each $i=1, 2, \dots , n$*.* Thus, the incumbent variation fitness landscape must take on minima at ${{\varvec{u}}}_{i} = {{\varvec{u}}}_{i}^{*}$ for each $i=1, 2, \dots , n$. In exploring the incumbent variation fitness landscape (NIS-landscape) in the vicinity of ${{\varvec{u}}}_{i}^{*}$ one must compute $G({{\varvec{u}}}_{i}^{*}, {{\varvec{u}}}^{*,i},\boldsymbol{ }{\varvec{x}}({{\varvec{u}}}^{*,i}))$ as ${{\varvec{u}}}_{i}$ is varied in the vicinity of ${{\varvec{u}}}_{i}^{*}$. Thus, the challenge in plotting the NIS-landscape is the need to constantly update the population sizes of all the species. In the case of single species evolution with scalar strategy, it has been shown that a singular point that is an ESS and NIS is also convergent stable. If not ESS, we conjecture that it is not possible for 2 species to coexist around that peak. It is interesting to note that mutual invasibility can occur even if the singular point is neither ESS, NIS nor convergent stable (Fig. 2h in [20]). Theory also suggests a singular point can be NIS but not allow for mutual invasiblity (see Fig. 2d) in [20]). Even in the case where the singular point is an ESS, NIS and thus convergent stable, additional condition needs to be met to guarantee mutual invasibility. It does follow that if the singular point is ESS and permits mutual invasiblity, then the point is both NIS and convergent stable. If it is NIS, then we conjecture it is possible as a necessary condition, but possibly not sufficient.

Vector-valued strategies create greater opportunities for hypersaturated communities than scalar strategies, yet understanding when and how non-ESS species can coexist becomes more challenging. Furthermore, rigorous mathematical characterizations do not yet exist beyond a single strategy ESS [18]. For instance, with scalar-valued strategies, two species can coexist (whether ESS or otherwise) if and only if there is at least one peak or valley of the adaptive landscape between them. In the example explored here, the hypersaturated community contained 3 species distributed around the single species ESS. Any of these two species can coexist without the third, and when they do there is generally not a peak or valley between them. Rather, if one examines the adaptive landscape as the plane between them there is always peak between them. We conjecture that this result will be necessary for pairwise coexistence. But will it be sufficient?

Once there are several species at the ESS in a bivariate strategy model then potentially there may be 3 times as many coexisting species in the hypersaturated community. However, like the 5-species ESS with scalar-valued strategies, diffuse competition and the lack of NIS-like properties may preclude achieving this maximum number. A rigorous characterization of convergence stability, NIS and mutual invasibility have not been achieved as of yet.

Here, we took advantage of the proof of Durinx et al. [18] that applies when there is just one species at the ESS. We explored to what extent it naturally extends to ESSs with multiple species. We saw that there are limits, which we hypothesized were a result of increasingly diffuse competition and lack of NIS or NIS-like properties. Our work also builds upon Rubin et al. [37] who demonstrated hypersaturated communities by starting with extreme numbers of species and letting them triage down to diverse communities of coexisting species at numbers well above the ESS diversity of species. Such communities can be defined as invasion structured. Here, we took a more fine-tuned approach in examining a few examples to generate specific conjectures regarding the properties of non-ESS communities be they under- or hypersaturated.

This, and related modelling work, raises important empirical questions. First, what are the differences in the community structure of invasion structured versus communities at ESSs? The latter, as has been shown, can have more or fewer species than the ESS. They may be ecologically stable but they are not evolutionarily so. Over time the evolved traits of these species can be expected to change. How much and how fast becomes the empirical question that determines the relevance of this and similar work to natural systems. When at ESS, the community is both ecologically and evolutionarily stable. Such communities are diverse enough as to be uninvadable and not so diverse as to be unstable. Second, to what extent are most natural communities at or near their ESS? And, if non ESS, are natural communities under-or hyper saturated?

Invasive species around the globe represent an important context for examining ESS and non-ESS communities. In one sense, ESS communities might be seen as the gold standard for conserving species and communities [27]. Within the present modeling framework, a community is susceptible to invasion by novel species for one of two reasons. Firstly, the invading species may represent a novel *G*-function, namely a species with a strategy and strategy set different from any of the species present in the recipient community [35]. Such is beyond the scope of our current modelling, but the modelling of communities with different *G*-functions such as predator–prey systems offer diverse pathways towards species coexistence and niche coevolution [36]. Secondly, the invading species may share the same *G*-function as the recipient community. In this case, it means the recipient community was not at its ESS. It could be that the invader falls into an invasion window, in which case it may take an undersaturated community and move it closer to the diversity of the ESS. Or, none of the resident species are at their peaks and the invader will either replace one of these species or contribute to a hypersaturated community.

The current bird communities of the Hawaiian Islands likely represent an example of both processes. Many of the bird Families that have been introduced from Asia, South America, Europe and North America represent novel “evolutionary technologies” (e.g., *G*-functions) not yet present in Hawaii. Kotler et al. [27] make the case that the taxonomic level of either Family or Order provides likely cut-offs for different *G*-functions (also see [7]). Of Hawaii’s roughly 64 endemic bird species, roughly 33 have gone extinct. At least 58 exotic species were able to establish large populations in Hawaii, and over time some have gone extinct themselves, leaving a current list of 52 well-established exotic species. The overall bird diversity of Hawaii has increased, likely as a result of new *G*-functions, the filling of unoccupied niches of under-saturated communities, and possibly creating non-ESS hypersaturated communities. One certainty is that the current birds of Hawaii form non-ESS, invasion structured communities.

The birds of Hawaii are a microcosm for how global climate change, invasive species, and land use changes likely leave most communities shifted away from their ESS. As non-ESS communities, we can expect to see eco-evolutionary dynamics result in species deletions for hypersaturated communities and additions to undersaturated ones. Rapid evolution has now been widely documented in human dominated landscapes [22, 26, 34, 38]. We see the modelling and understanding of ESS and non-ESS communities as essential to anticipating these changes rather than being bystanders.

## Supplementary Information

Below is the link to the electronic supplementary material.

Supplementary file1 (DOCX 1631 KB)

## Data Availability

No datasets were generated or analysed during the current study.
