# Supplementary material for: Hyper Diversity, Species Richness, and Community Structure in ESS and Non-ESS Communities
Source: Dyn Games Appl. 2025 May 30;15(4):1424–44. doi: 10.1007/s13235-025-00646-2 (PMC12460531; doi:10.1007/s13235-025-00646-2)
Supplement: Supplementary file 1 — Supplementary file1 (DOCX 1631 KB) [file 13235_2025_646_MOESM1_ESM.docx]

**Supplementary material**


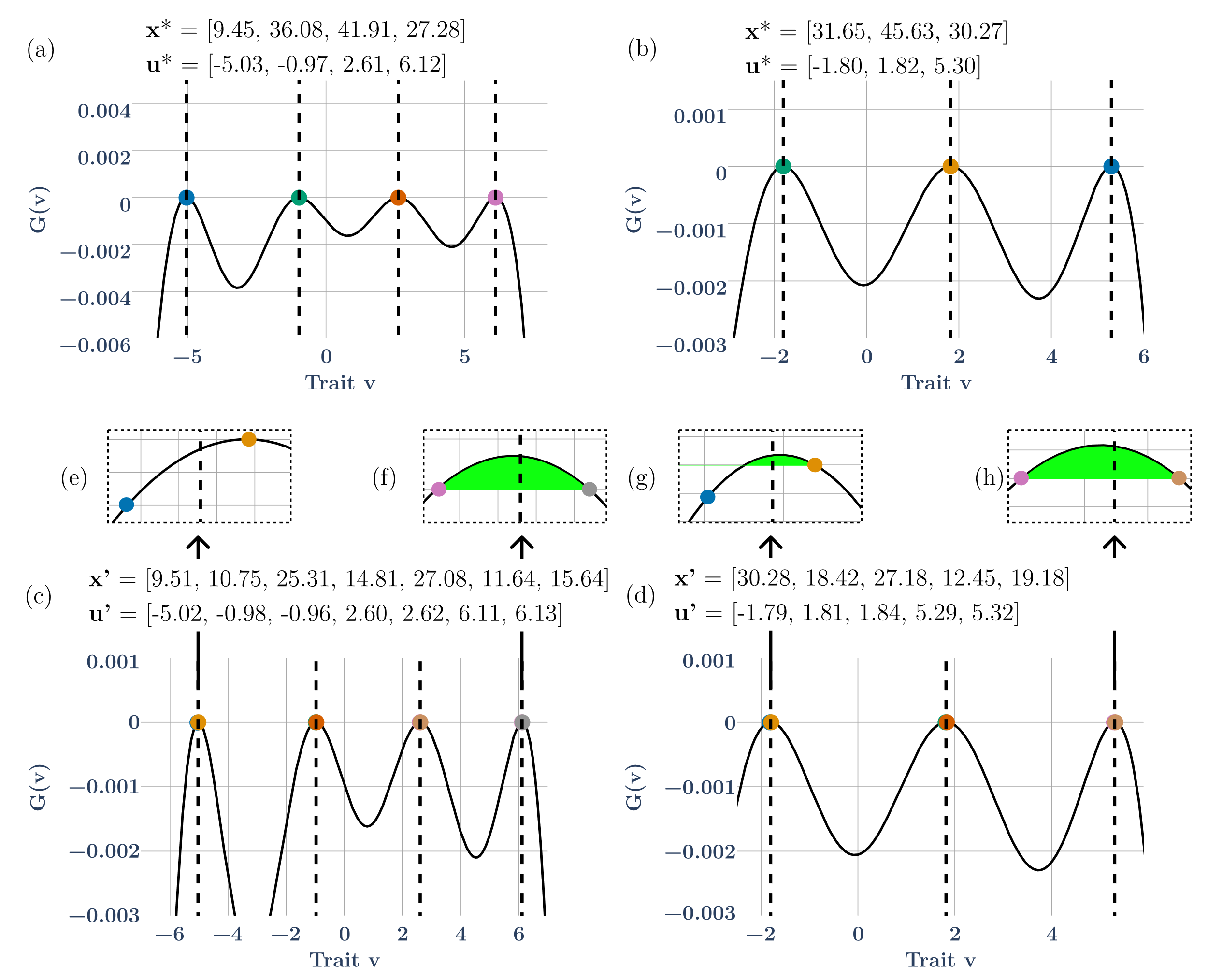


Fig S 1 The 3 and 4 species ESS cases (a, b, respectively). (c) and (d) show 5-species and 7-species non-ESS communities respectively. Vertical dotted lines mark the ESSes. Straddling two species around the first peak in both cases always led to one of them having negative fitness (insets (e), (g), blue species does not survive) and hence going extinct, while species around other peaks survive and coexist (insets (f), (h)). Here $\sigma_{K}^{2}$ values are 30, and 40 respectively for the 3-species ESS and the 4-species ESS.

**Species removal in 5-species non-ESS coexistence:**

The 3-ESS peaks give rise to 5 non-ESS populations that can coexist. Individual species removal exercises was conducted on this system. Removing species 5 (highest strategy value) causes species 3 to go extinct, and releases the others, while removing species 4 causes species 2 to go extinct. Removing species 2 is inconsequential as the other four species can coexist. Absence of species 3 however leads to extinction of species 4. Removing species 1 causes species 3 and 4 to go extinct and species 2 and 5 experience competitive release.

**Species removal in 7-species non-ESS coexistence:**

The 4-ESS peaks give rise to 7 non-ESS populations that can coexist. Removal of species 7 (highest strategy value) results in species 5 going extinct and species 4 and 6 experiencing competitive release. Removing species 6 causes species 4 to go extinct and release of species 5 and 7. The absence of species 5 leads to extinction of species 3, and species 2 and 4 experience competitive release. Species 4 being removed causes species 2 to go extinct, and species 5 and 3 are released. Removing species 3 leads to the extinction of species 4 and release of species 2 and 5. Removal of species 2 leads to stable coexistence of the other species and removal of species 1 causes species 2, 5 and 6 to go extinct while the rest are released.

Thus, the 7-species non-ESS community in the 4-species ESS case is more sensitive to species removal than the 7-species non-ESS community in the 5-species ESS case (Fig 5 (b) vs Fig S2)


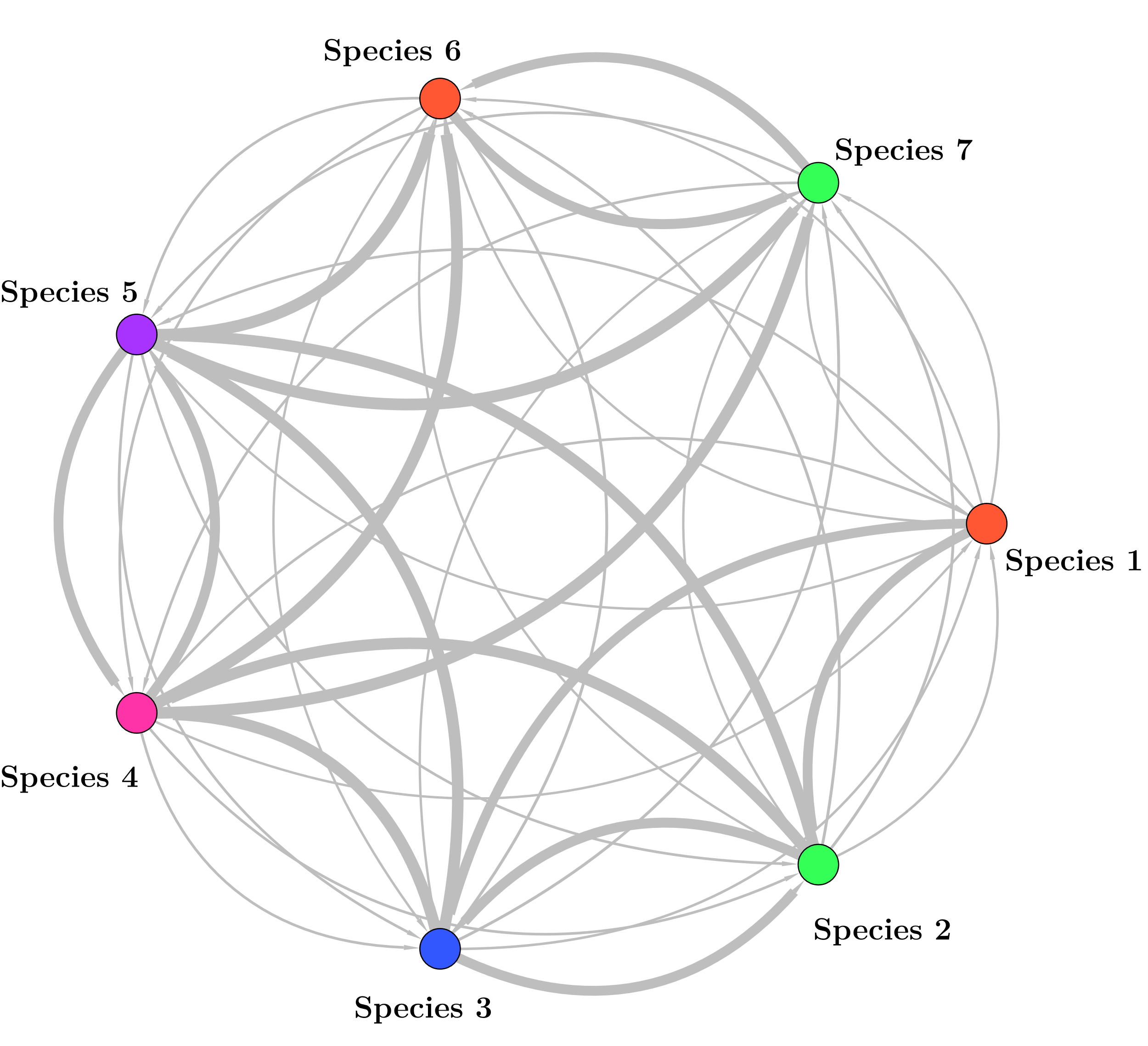


Fig S 2: Interaction network for the 7-species non-ESS community as a result of straddling each of the 4-species ESSes in Fig S1.

**Species diversity at ESS for different combinations of** $\boldsymbol{\sigma}_{\boldsymbol{\alpha}}^{\boldsymbol{2}}$ **and** $\boldsymbol{\sigma}_{\boldsymbol{K}}^{\boldsymbol{2}}$**:**

Since the number of species at the ESS depend on the values of $\sigma_{\alpha}^{2}$ and $\sigma_{K}^{2}$, we performed a grid search for $\sigma_{\alpha}^{2}$ in the range [4, 21] and $\sigma_{K}^{2}$ in the range [4, 104] with a step size of 1. We seeded $50$ populations with initial trait values $\boldsymbol{u}^{0}$ uniformly spaced in the interval [-15, 15], and initial population sizes $\boldsymbol{x}^{0}$ randomly chosen from the interval [$\frac{K_{max}}{125},\frac{K_{max}}{100}$] where $K_{max}=100$ is the maximum carrying capacity. The system was then allowed to reach both ecological and evolutionary equilibria following the dynamics given by equations Eq 1 and Eq 2 (main text). For our numerical solutions, ecological and evolutionary equilibria are said to be reached when both the change in frequencies of the populations and change in trait values $\boldsymbol{u}$ of the populations are less than ${10}^{-7}$ in 5000 simulated time units respectively (Eq 9 and Eq 10 in the main text). Due to the randomness in choosing initial population sizes, the number of species at ESS varied in different runs. As these simulations are computationally expensive, we only ran 15 simulations. Fig S3 shows the most frequently occurring number of species at ESS for each combination of $\sigma_{\alpha}^{2}$ and $\sigma_{K}^{2}$.


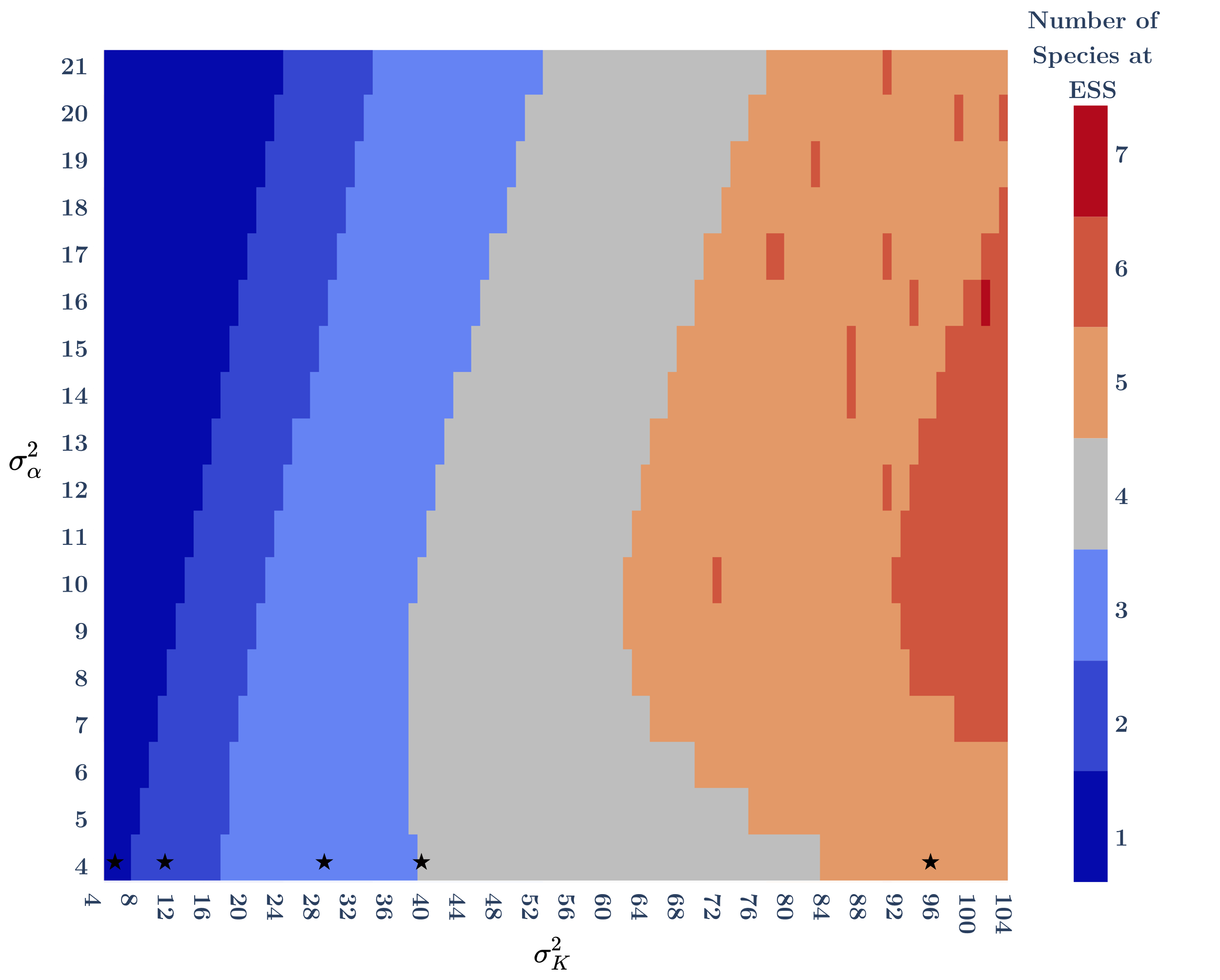


Fig S 3 Heatmap showing the number of species at the ESS for different combinations of the parameters $\sigma_{\alpha}^{2}$ and $\sigma_{K}^{2}$. The stars mark the parameter ($\sigma_{\alpha}^{2}$, $\sigma_{K}^{2}$) combinations used in this study to achieve 1, 2, 3, 4, and 5 species ESSes. Since the method of finding the ESSes uses random initial population sizes, the number of species that survive once ecological and evolutionary equilibrium are reached may vary across runs, leading to some parameter combinations within the 5-species ESS region (light orange) having 6-species ESSes (dark orange).
